# Sputtered Modified Barium Titanate for Thin-Film Capacitor Applications

**DOI:** 10.3390/ma5040575

**Published:** 2012-04-10

**Authors:** Glyn J. Reynolds, Martin Kratzer, Martin Dubs, Heinz Felzer, Robert Mamazza

**Affiliations:** 1 Oerlikon USA, Inc., Business Unit Systems, 970 Carillon Dr., Suite 300, St. Petersburg, FL 33716, USA; 2 OC Oerlikon Balzers AG, Business Unit Systems, Iramali 18, P.O. Box 1000, LI-9496 Balzers, Liechtenstein; E-Mails: martin.kratzer@oerlikon.com (M.K.); martin-dubs@bluewin.ch (M.D.); heinz.felzer@oerlikon.com (H.F.); robert.mamazza@oerlikon.com (R.M.)

**Keywords:** RF sputtering, barium titanate, high-k

## Abstract

New apparatus and a new process for the sputter deposition of modified barium titanate thin-films were developed. Films were deposited at temperatures up to 900 °C from a Ba_0.96_Ca_0.04_Ti_0.82_Zr_0.18_O_3_ (BCZTO) target directly onto Si, Ni and Pt surfaces and characterized by X-ray diffraction (XRD), scanning electron microscopy (SEM) and X-ray photoelectron spectroscopy (XPS). Film texture and crystallinity were found to depend on both deposition temperature and substrate: above 600 °C, the as-deposited films consisted of well-facetted crystallites with the cubic perovskite structure. A strongly textured Pt (111) underlayer enhanced the (001) orientation of BCZTO films deposited at 900 °C, 10 mtorr pressure and 10% oxygen in argon. Similar films deposited onto a Pt (111) textured film at 700 °C and directly onto (100) Si wafers showed relatively larger (011) and diminished intensity (00ℓ) diffraction peaks. Sputter ambients containing oxygen caused the Ni underlayers to oxidize even at 700 °C: Raising the process temperature produced more diffraction peaks of NiO with increased intensities. Thin-film capacitors were fabricated using ~500 nm thick BCZTO dielectrics and both Pt and Ni top and bottom electrodes. Small signal capacitance measurements were carried out to determine capacitance and parallel resistance at low frequencies and from these data, the relative permittivity (ε_r_) and resistivity (ρ) of the dielectric films were calculated; values ranged from ~50 to >2,000, and from ~10^4^ to ~10^10^ Ω∙cm, respectively.

## 1. Introduction

In a review published in 2000, Bhalla *et al.* described barium titanate and its relatives with the perovskite structure as “the most significant electroceramic dielectric phase in industry” and discussed how changing the composition could have noteworthy consequences for many important applications [1]. In the same year, a US Patent was issued to Hansen that claimed a family of materials based on barium titanate with extremely high relative permittivities, ranging from ~20,000 to over 33,000 [2]. Since then, similarly doped barium titanates have found extensive application in multi-layer ceramic capacitors (MLCC), used widely in the electronics industry [3]. Capacitors based on these modified barium titanates have even been proposed for vehicle propulsion applications [4,5,6].

Today, most MLCCs are fabricated using thick film techniques such as tape casting [7]. However, the future needs of the industry require capacitors with very thin layers that are not easily fabricated with these techniques [8]. In addition, there has been considerable recent interest in System-in-a-Package (SiP) semiconductor products that incorporate integrated passive devices such as resistors, inductors and capacitors deposited either directly onto an integrated circuit prior to or during packaging, or onto a silicon or glass substrate on which many different components are mounted [9,10,11]. These particular applications lend themselves to thin-film techniques that are already well-established in the semiconductor industry, for example, sol-gel deposition, chemical vapor deposition (CVD) and physical vapor deposition (PVD), and that are capable of depositing thinner layers than currently possible by typical thick film methods. Each of these thin-film deposition techniques has advantages and disadvantages. Excellent film properties have been reported for films deposited by sol-gel but this method is relatively slow and labor intensive [12,13,14,15,16]. CVD methods provide for conformal deposition into high aspect ratio features that can maximize electrode-to-dielectric surface area thus providing for increased capacitance in a given unit area but they require a cocktail of expensive and often toxic or flammable precursors [17,18,19,20]. If suitable target materials are available, radio frequency (RF) magnetron sputtering can provide a cost-effective, productive and clean approach to thin-film capacitor dielectric deposition [21,22,23,24,25].

Co-sputtering from multiple targets in a single vacuum chamber has the potential to quickly and efficiently deposit closely-related materials with a wide range of composition. This paper describes a novel high temperature sputtering apparatus capable of depositing doped barium titanates in the desired perovskite structure and on the characterization of the thin-films so produced.

## 2. Experimental Section

### 2.1. Apparatus

**Figure 1:**
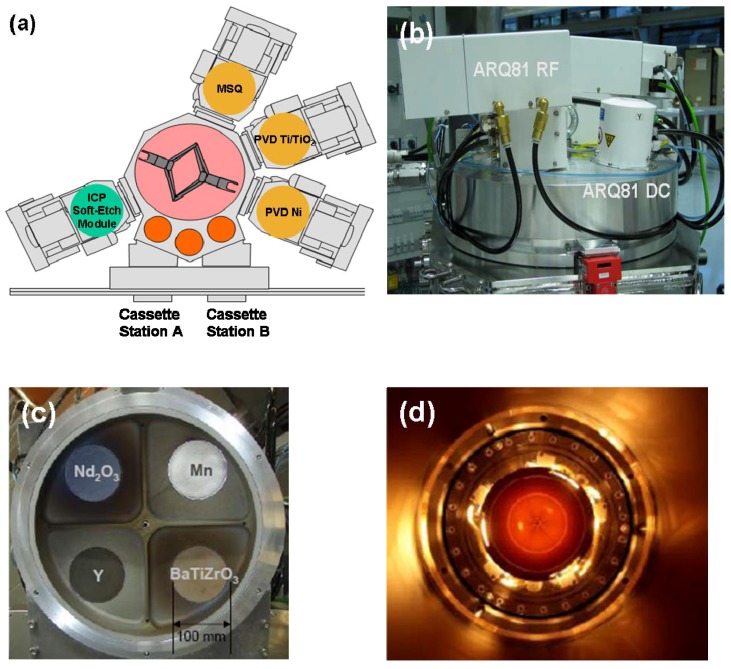
**(a)** Schematic of Oerlikon ClusterLine 200 II used for this work. **(b)** External view of Oerlikon MSQ showing independently powered sources, two RF, two pulsed DC. **(c)** Internal view of multiple targets and source shield. **(d)** Top down view of very hot rotating chuck at temperature.

An Oerlikon ClusterLine 200 II cluster tool, a fully-automated, computer-controlled system was used for this work. It comprised a central transfer module surrounded by two vacuum load locks and four process modules; additional auxiliary modules performed alignment, wafer pre-heat and wafer cool functions and a robotic arm transferred the wafers between modules under vacuum (Figure 1a). 

Prior to deposition, wafers were sputter etched in order to remove surface contamination. This was done in a chamber that combined a low frequency RF inductively coupled plasma with 13.56 MHz wafer bias on the substrate. Two standard PVD modules, one equipped with a Ti target and a pulsed DC power supply, and the other equipped with a Ni target and a standard DC power supply were used to deposit Ti/TiO_2_ bilayers and the Ni electrodes, respectively. These modules used a weighted clamp and backside argon to promote conductive coupling between the wafer and a temperature-controlled, heated chuck.

Both Ni and Pt bottom electrodes were used in this work. Ni was sputtered in the tool in the Oerlikon Systems R&D laboratory; Pt was deposited in a similar system located in the Army Research Laboratory (ARL), Adelphi, Maryland. The ARL tool was also equipped with a PVD module with a Ti target so that Ti/TiO_2_ bilayers could be deposited prior to sputtering the Pt bottom electrodes.

The apparatus used to sputter deposit the modified barium titanate dielectric was designed and built purposely for this work and will be described in more detail here (see Figure 1b). The Multisource Quattro (MSQ) is a co-sputtering source that can be configured with up to four 100 mm diameter targets (Figure 1c). Also visible in this figure is a shield specially designed to obstruct line-of-sight between each of the four targets, thereby reducing cross-contamination between them. Two of the sources are capable of RF sputtering: this was required because the modified barium titanate targets used here are insulators. The remaining two sources have pulsed DC power supplies, enabling them to reactively sputter metallic targets in oxygen- or nitrogen-containing ambients. The substrate was supported on a very hot rotating chuck capable of achieving temperatures above 900 °C (Figure 1d). Backside argon gas was provided to improve conductive coupling between chuck and substrate and a weighted clamp ring held the latter in place during processing.

An automated co-sputtering source is capable of depositing films with a wide range of stoichiometry *in-situ* without the need for many different target formulations. The four targets available on the source used here allow the independent controlled addition of up to three dopants to a standard film composition. The material found by Hansen to have the highest ε_r_ value had the overall stoichiometry [Ba_0.9575_Nd_0.0025_Ca_0.04_][Ti_0.815_Mn_0.0025_Y_0.0025_Zr_0.18_]O_3_ [2] and this stoichiometry was also chosen by Weir and Nelson as the primary component in the dielectric of their “Electrical Energy Storage Unit” (EESU) [4,5,6]. The added Group III and VII elements can act as donors and acceptors in the perovskite lattice. The Multisource used for this work was configured with a target containing added Ca and a fixed Ti:Zr ratio identical to Hansen’s material, plus additional targets of Mn, Y and Nd_2_O_3_ that would allow the concentrations of these dopants to be varied. This paper describes only capacitors fabricated with the base Ba_0.96_Ca_0.04_Ti_0.82_Zr_0.18_O_3_ (BCZTO) dielectric material.

### 2.2. Deposition

Capacitor structures were fabricated as follows: first, the bottom electrodes were sputter deposited onto 4-inch Si or oxidized Si wafers. The preferred bottom electrode was Ni but additional Pt bottom electrodes were sputter deposited at ARL. It was found to be especially advantageous to deposit thin bilayers of Ti/TiO_2_ below the Pt: the Ti layer promotes adhesion between the Pt and the substrate and the TiO_2_ acts both to prevent attack of the Ti adhesion layer by any oxygen that diffuses through the Pt during reactive sputter deposition of the dielectric films and also to prevent interaction between the Ti, O and Pt [26]. Ti/TiO_2_ bilayers were also used below many of the Ni bottom electrodes.

The BCZTO dielectric films were deposited over a range of conditions listed in Table 1. Temperature was controlled by applying a given power to the tungsten halogen lamps below the rotating chuck: wafer temperature *vs.* lamp power was calibrated by pyrometry using a wafer of known emissivity. Wafers were introduced with the rotating chuck already at process temperature and usually allowed to remain on the chuck for 15 minutes to reach thermal equilibrium prior to starting the deposition. Typical BCZTO film thicknesses were ~500 nm: Depending on the sputtering conditions employed and film thickness desired, process times were usually in the range 3–8 hours.

**Table 1:** Deposition conditions and rates for BCZTO sputtered thin-films.

Deposition temperature (°C)	Target power (W)	Sputter pressure (mtorr)	% O_2_ in Ar	Rate (nm/s)
700	250	4.5	0	0.0458
700	250	10.0	0	0.0394
700	250	4.5	10	0.0184
700	250	10.0	10	0.0163
700	250	4.5	15	0.0169
700	250	10.0	15	0.0145
800	250	7.25	5	0.0174
900	250	4.5	0	0.0454
900	250	10.0	0	0.0420
900	250	4.5	10	0.0185
900	250	10.0	10	0.0156
900	250	4.5	15	0.0168
900	250	10.0	15	0.0132

**Figure 2:**
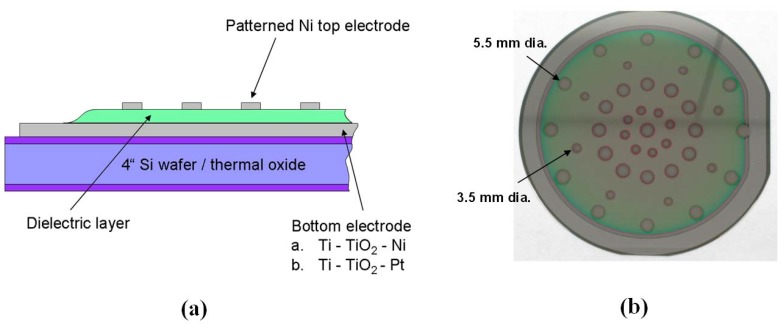
**(a)** Schematic cross-section of thin-film capacitor structure. **(b)** Processed wafer after sputter deposition of Ni top electrode dots through a shadow mask.

Pt or Ni was used for the top electrodes. After depositing the dielectric, the wafer was allowed to cool *in vacuo* and Ni was deposited through a shadow mask. The mask was machined to have concentric rings of two different sized holes that produced ~3.5 mm and ~5.5 mm diameter circular Ni dots on the wafer. The former gave a more realistic estimate of the breakdown voltage and leakage currents intrinsic to the dielectric films, since the smaller area lessened the probability of a defect in the capacitor dielectric; the larger capacitors allowed a better estimate of the specific energy. A schematic of this structure is shown in Figure 2a; Figure 2b shows a picture of a processed wafer.

Some wafers were sealed under N_2_ and shipped to ARL where a blanket Pt film was first sputter deposited and then patterned into 1 mm top electrode squares using lithography and ion milling.

### 2.3. Characterization

Film thickness was measured by profilometry and for films thinner than 200 nm, by X-ray reflectometry (XRR). The latter served as a useful cross-check for profilometry measurements. X-ray diffraction (XRD) and X-ray reflectometry (XRR) measurements were performed at NTB Buchs, Switzerland; depth profile X-ray photoelectron spectroscopy (XPS) was performed by the Evans Analytical Group, East Windsor, New Jersey; scanning electron microscopy (SEM) was performed in-house and at EMPA St. Gallen, Switzerland; and profilometry was performed in-house. Electrical characterization was done in-house and at ARL.

## 3. Results and Discussion

### 3.1. XRD and SEM

X-ray diffraction data were obtained using a Material Research Diffractometer X-Pert (MRD-XL) (Fa. PANalytical B.V., Netherlands) and Cu Kα radiation. For experiments using a grazing incidence configuration, the incidence angle was 0.9°.

**Figure 3:**
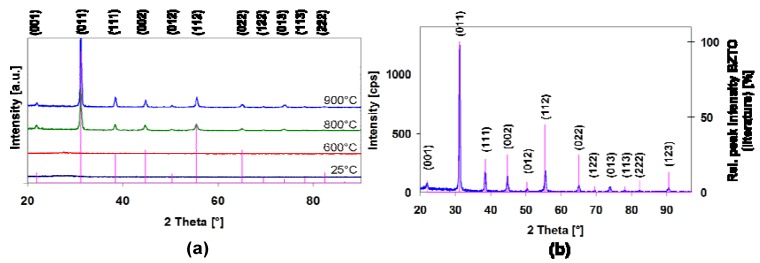
**(a)** Grazing incidence XRD data (Cu Kα radiation) for a series of BCZTO thin-films deposited on Si (100) between ambient temperature and 900 °C. All films were deposited at 4.5 mtorr using Ar only. **(b)** Shows peaks for film deposited at 900 °C indexed to cubic barium zirconate-titanate using the powder diffraction database [27].

Figure 3a shows the grazing incidence XRD patterns of a series of BCZTO films deposited onto Si (100) over a range of temperatures between ambient and 900 °C at 4.5 mtorr with Ar only. No diffraction peaks were observed at deposition temperatures ≤600 °C suggesting that these films were either amorphous or nanocrystalline. Films deposited above 600 °C showed only the characteristic peaks of cubic barium zirconate-titanate. No additional peaks that could be assigned to any crystalline impurities were observed: typically, this indicates that the films were >95% pure. Figure 3b shows in more detail the pattern of the BCZTO film deposited at 900 °C indexed based on the cubic perovskite structure of barium zirconate-titanate. Although this phase is metastable at ambient temperature, it is thermodynamically stable at the temperature of deposition. Rapid cooling through the Curie point likely freezes in the cubic structure.

**Figure 4:**
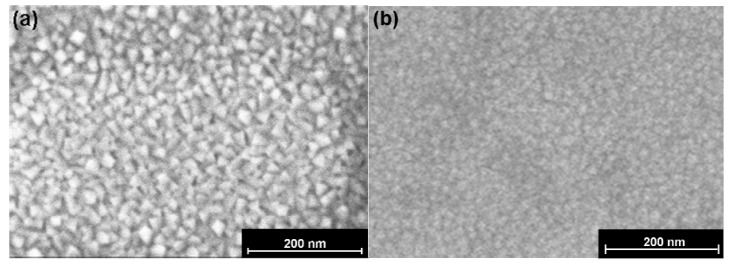
**(a)** SEM view from above of BCZTO film deposited at 800 °C. **(b)** SEM view from above of BCZTO film deposited at ambient temperature. Note the well facetted crystals in **(a)**.

Figure 4a,b shows the scanning electron micrographs (SEMs) of the surfaces of two different modified barium titanate thin-films sputtered onto Si wafers, one at 800 °C (Figure 4a) and the other at ambient temperature (Figure 4b). Note the significant contrast in surface structure: While Figure 4a clearly shows facetted crystals, Figure 4b is essentially featureless.

**Figure 5:**
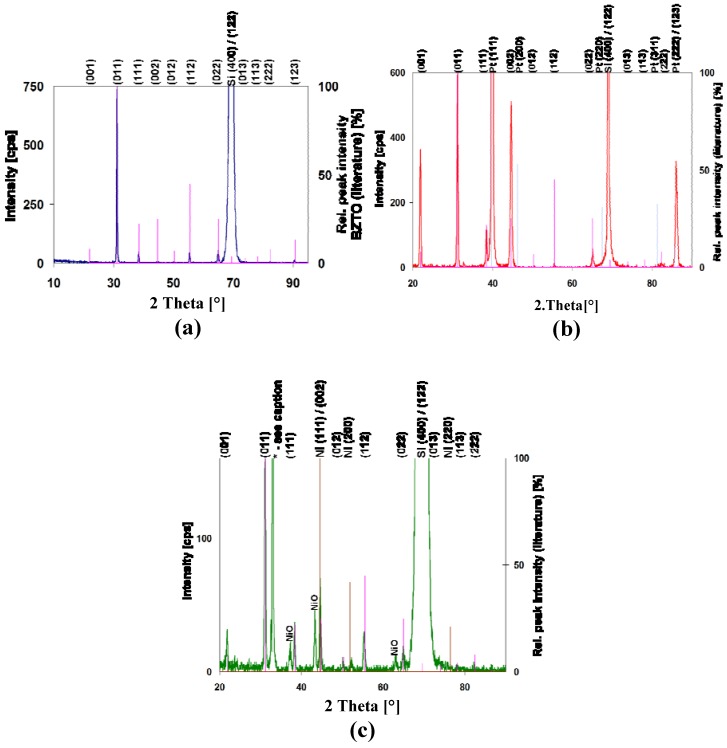
θ–2θ XRD pattern (Cu Kα radiation) of BCZTO films deposited at 900 °C, 10 mtorr pressure, 10% O_2_ in Ar onto: **(a)** a bare (100) Si wafer; **(b)** a Pt bottom electrode structure; **(c)** a Ni bottom electrode structure. Peak intensities are normalized to the BCZTO (011) peaks at 100% relative intensity. Also shown for reference are the peak positions and relative intensities of barium zirconate-titanate, Pt and Ni taken from the powder diffraction database [27,28,29] *.

Figure 5 compares the θ–2θ XRD patterns of a BCZTO film deposited at 900 °C, 10 mtorr with 10% O_2_ in Ar on bare Si (100) with those deposited at similar conditions on Pt and Ni bottom electrode structures. Both electrode films were deposited over Ti/TiO_2_ underlayers. Also shown for reference are the peak positions and intensities of barium zirconate-titanate, Pt and Ni from the powder diffraction file [27,28,29]. All peak intensities are normalized to a BCZTO (011) peak intensity of 100%. Note that both Pt and Ni electrode films exhibit strong (111) preferred orientation.

On bare Si (100), BCZTO appears to exhibit preferred (011) orientation: note the absence of (001) and (002) reflections. In contrast, on Pt the intensities of (001) and (002) reflections are increased relative to (011). On Ni, there is little evidence for preferred orientation of BCZTO: the proximity of the Ni (111) peak at ~44.5° and the BCZTO peak at ~44.7° makes it difficult to determine whether there is enhancement of the (00ℓ) reflections, though the slight enhancement of the (001) peak compared to the powder data may provide some slight evidence for this. Overall, the peak intensities of BCZTO on Ni are lower suggesting a smaller grain size or a less crystalline film. Perhaps more significantly, the peaks of NiO [30] are clearly visible at ~37.2°, ~43.3° and ~62.9° indicating that the Ni bottom electrode is oxidized during the high temperature sputter deposition of BCZTO in Ar/O_2_.

**Figure 6:**
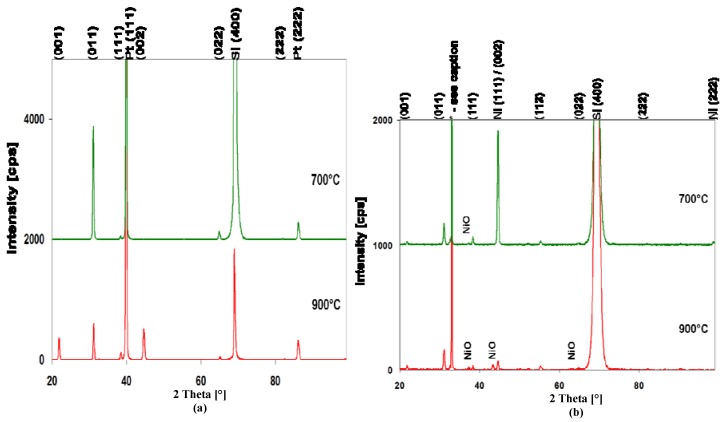
θ–2θ XRD pattern (Cu Kα radiation) of BCZTO films deposited onto: **(a)** Pt (111) and **(b)** Ni (111) bottom electrodes at 700 °C (upper traces) and 900 °C (lower traces) (10 mtorr pressure, 10% O_2_ in Ar, Si (100) substrate in all cases).

The effect of changing the deposition temperature on the diffraction pattern of BCZTO films on Pt and Ni is shown in Figure 6. On Pt, decreasing the deposition temperature from 900 °C to 700 °C increases the relative intensity of the BCZTO (011) peak while the (001) and (002) peaks disappear. On Ni, while the relative BCZTO peak intensities remain essentially unchanged, decreasing the deposition temperature from 900 °C to 700 °C decreases the number and intensity of NiO peaks observed. It is to be expected that the rate of oxidation of the Ni bottom electrode would be slower at lower BCZTO deposition temperatures. However, even at 700 °C, a Ni electrode exposed to a mixture of 10% O_2_ in Ar for 60 minutes was oxidized, showing diffraction peaks of NiO. At 700 °C, the growing film of BCZTO on top of the Ni electrode may serve either to reduce the effective fugacity of oxygen at the Ni surface or to reduce the rate of oxygen diffusion, thereby limiting the amount of NiO formed at the interface.

* Other authors have observed the peak appearing at ~32.9° in Figure 5c and Figure 6b and have determined that it is caused by multiple scattering rather than by Si (200) lattice planes that could give rise to a peak at this 2θ value if it were not forbidden for the Fd3m space group [24].

### 3.2. XPS

**Figure 7:**
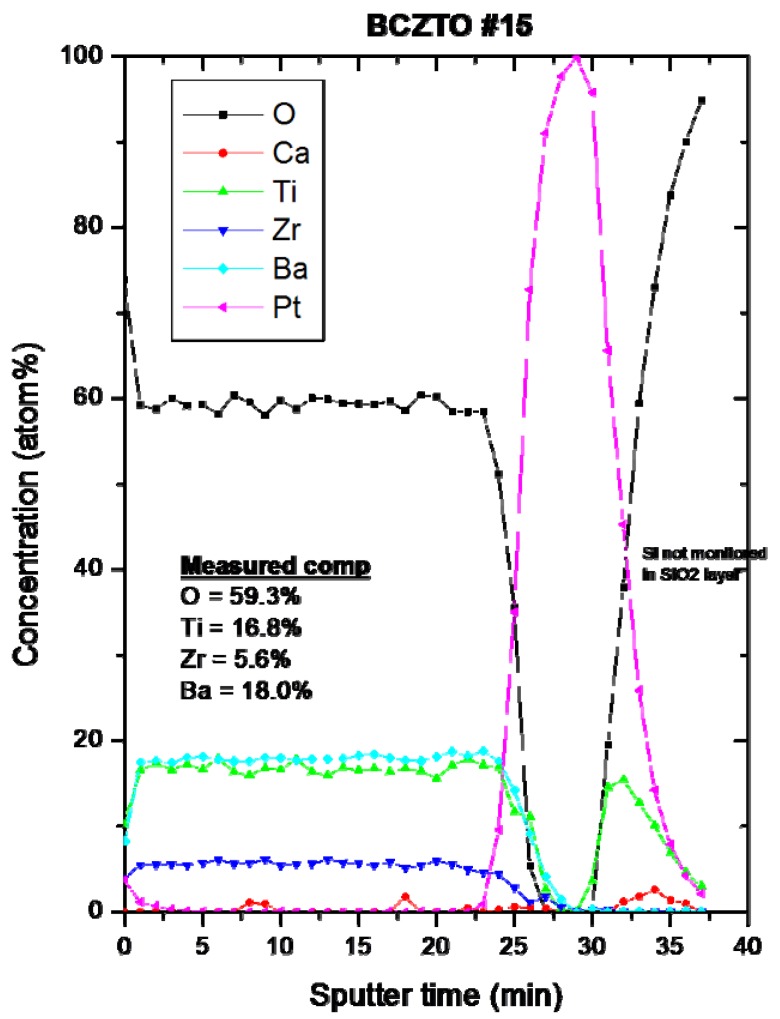
Depth profile XPS of ~500 nm thick BCZTO film with Pt electrodes. Sample area was ~600 μm square, located between patterned top electrode dots.

A ~458 nm thick sample (as determined by profilometry) of BCZTO deposited on an oxidized 4-inch Si wafer with Pt top and bottom electrodes in place was analyzed by depth profile XPS analysis using a PHI Quantum 2000 instrument, monochromated Al Kα radiation at 1486.6 eV, an acceptance angle of ± 23° and a take off angle of 45°; the area of analysis was 600 μm square and was located between the 1 mm square dots that served as the top electrodes. The data are shown in Figure 7: the overall measured stiochiometry is close to the nominal composition of the target (Ba 19.2%, Ca 0.8%, Ti 16.4%, Zr 3.6% and O 60%) except that no firm evidence of Ca was seen in the film. This discrepancy might possibly be caused by a known interference between Zr and Ca: the fact that the Zr content is a little higher than expected supports this explanation. However, at 0.8 at.%, the amount of Ca in the film is close to the detection limits of the equipment (between 0. 05 and 1.0 at.%) and since Ca is one of the lighter elements in the film, the detection levels are apt to be towards the high end of this range. It is also possible that the Ca concentration in the film was lowered due to preferential scattering of the Ca in the sputtering ambient or by formation of Ca-containing compounds with significant vapor pressures at the deposition temperatures used here.

### 3.3. Electrical Characterization

The capacitance and parallel resistance of thin-film capacitors with ~500 nm thick dielectrics were measured at small signal (50 mV, 20 Hz) using an Agilent 4284A Precision LCR meter with a test signal frequency range of 20 Hz–1 MHz. From these results the relative permittivities and resistivities of the modified barium titanate films were calculated and are shown for different electrode materials and dielectric deposition conditions in Table 2. Also shown are selected loss factor data for capacitors with Pt bottom and Ni top electrodes.

All capacitors tested exhibited high leakage currents and for some deposition conditions, the dielectric layer was too conductive to obtain a reliable capacitance measurement. Many of the resistivities observed here are typical of semiconducting materials; even the best (most resistive) films would be classified as “leaky insulators”. Time constants (R·C) for the best capacitors measured here at small signal were in the range 0.3–0.5 s.

There was a significant difference in capacitance between devices with a Ni and a Pt bottom electrode if oxygen was added to the sputter ambient. This was attributed to oxidation of the Ni when exposed to oxygen at high temperatures. The semi-insulating nickel oxide that forms at the interface between the Ni bottom electrode and the BCZTO dielectric is thought to act as a capacitance in series —this leads to an effective reduction of the overall capacitance of the device:

(1) *C_eff_* = *C_BCZTO_* · *C_IF_*/(*C_BCZTO_* + *C_IF_*)

where C_eff_ is the effective capacitance, C_BCZTO_ is the capacitance due to the BCZTO dielectric and C_IF_ is the additional capacitance due to the oxidized Ni film at the interface. Experiments to prevent oxidation of the Ni bottom electrodes by: (i) omitting the temperature stabilization step prior to BCZTO deposition; (ii) depositing the first 30 nm layer of BCZTO without oxygen in the sputter ambient; and (iii) depositing the first 30 nm layer of BCZTO at 700 °C (instead of 900 °C) did little to suppress NiO formation at the electrode-dielectric interface.

Oxygen vacancies can act as electron donors in barium titanate and thereby lead to an increase in electrical conductivity [31]. Increasing the O_2_ fraction to 15% slightly increased the BCZTO resistivity at 700 °C but at 900 °C, results were similar to films deposited with 10% O_2_ (see Table 2).

**Table 2:** Selected electrical properties (50 mV, 20 Hz) of BCZTO thin-film capacitors as a function of electrode material and dielectric deposition conditions.

Bottom electrode	Top electrode	Dielectric T_dep_ (°C)	Process pressure (mtorr)	% O_2_ in Ar	ε_r_	ρ (Ω∙cm)	Loss factor
**Pt**	**Pt**	700	4.5	0		6.1 E+03	
		700	10	0		2.0 E+04	
		700	4.5	10	633	2.7 E+09	
		700	10	10	596	2.4 E+09	
		800	7.25	5	1006	2.8 E+09	
		900	4.5	0		2.2 E+05	
		900	10	0	899	4.0 E+09	
		900	4.5	10	1213	2.3 E+09	
		900	10	10	1023	4.1 E+09	
**Pt**	**Ni**	700	4.5	0	2018	1.2 E+08	0.66
		700	10	0	845	5.5 E+08	0.14
		700	4.5	10	900	5.3 E+08	0.13
		700	10	10	867	2.5 E+08	0.23
		700	4.5	15	659	1.5 E+09	
		700	10	15	529	1.3 E+09	
		800	7.25	5	1149	5.6 E+08	0.17
		900	4.5	0		2.0 E+04	
		900	10	0	1280	2.0 E+09	0.03
		900	4.5	10	1359	2.0 E+09	0.03
		900	10	10	1327	2.5 E+09	0.017
		900	4.5	15	1212	3.2 E+09	
		900	10	15	1193	3.4 E+09	
**Ni**	**Ni**	700	4.5	0	1738	3.6 E+08	
		700	10	0	964	1.8 E+08	
		700	4.5	10	331	3.9 E+09	
		700	10	10	398	8.0 E+08	
		800	7.25	5	123	4.6 E+08	
		900	4.5	0		3.9 E+04	
		900	10	0	1664	3.2 E+08	
		900	4.5	10	100	1.8 E+09	
		900	10	10	144	3.3 E+08	

The introduction of O_2_ to the sputter gas mixture and higher process pressures produce lower overall deposition rates. Additional experiments were performed where a series of alternating BCZTO layers, each ~40–50 nm thick, were deposited at different conditions. The results are shown in Table 3. For the first two experiments, higher deposition rates than the reference process were observed with only a small effect on the overall resistivity. Even the third experiment that produced an overall drop in rate compared to the reference process had a ~10% higher rate than if the whole deposition had been performed at 10 mtorr with 10% O_2_ in Ar.

**Table 3:** Effect of sputtering alternating layers of BCZTO at different process conditions on electrical properties and deposition rates.

Bottom electrode	Top electrode		Dielectric T_dep_ (°C)	Process pressure (mtorr)	% O_2_ in Ar	ε_r_	ρ (Ω∙cm)	Dep. rate *vs.* reference
**Pt**	**Ni**	**Layer 1**	900	4.5	10	1091	3.4 E+08	**+42%**
		**Layer 2**	900	4.5	0			
		**Layer 1**	900	4.5	10	1131	2.5 E+09	**+35%**
		**Layer 2**	900	10	0			
		**Layer 1**	900	4.5	10	1261	2.7 E+09	**-9%**
		**Layer 2**	900	10	10			
		**Reference **	900	4.5	10	1359	2.0 E+09	**–**

### 3.4. Discussion

RF sputtering from a compact co-sputtering source proved to be a suitable method for depositing thin dielectric films. This type of apparatus can be set up to add dopants to a material such as barium titanate, thereby allowing convenient investigation of their effect on the relative permittivity and other electrical properties. Once an optimum film composition is identified, it is generally more productive to switch to a conventional sputtering chamber configuration with a single planar target owing to the higher deposition rates and better target utilizations typically associated with a standard arrangement.

The as-deposited BCZTO films investigated here were electrically leaky. This is probably due to the many oxygen vacancies incorporated during film growth. It is known from the literature that both the addition of dopants and the oxidizing or reducing nature of the elevated temperature firing conditions significantly affect the conductivity of barium titanate-based dielectric materials [32]. Additional experiments are required to determine the best approach for increasing the resistivity of these sputtered films. A high temperature heat treatment in a controlled pO_2_ oxidizing environment is compatible with inert Pt electrodes and will also serve to anneal out any sputtering damage created during deposition or patterning of the top electrode, while co-sputtering is ideally suited for the controlled addition of dopants. MLCCs with base metal electrodes such as Ni that are subject to oxidation at elevated temperatures require high temperature firing of the capacitor stack in forming gas or a CO/CO_2_ gas mixture to reduce any unwanted metal oxides present, followed by a lower temperature step in an oxygen-containing ambient to anneal out oxygen vacancies [33]; it is likely that thin-film capacitors whose dielectrics have been deposited at high temperature on Ni electrodes will require similar post-processing. For single layer thin-film capacitors, another viable option is to use a Pt bottom electrode and a Ni or Cr top electrode that is evaporated through a shadow mask after the dielectric has been deposited and subsequently annealed in an oxidizing ambient; this circumvents any problems associated with sputter damage of the dielectric or oxidation of base metals and could be an appropriate topic for further investigation.

## 4. Conclusions

Thin films of high-k dielectrics based on barium titanate were deposited in their desired perovskite structure by RF sputtering above 600 °C. Simple single layer capacitors were fabricated by sequentially depositing bottom electrode, dielectric and top electrode in a cluster tool without breaking vacuum. Film texture, crystallinity and electrical properties were shown to depend on the deposition conditions and underlying substrate. When exposed to oxygen-containing sputtering ambients at ≥700 °C, Ni bottom electrodes showed evidence of oxidation which resulted in lower measured capacitance values.

In a separate publication, additional electrical characterization data on the breakdown voltage (V_BD_), leakage currents, polarization and specific energy of these thin-film capacitors is reported [34]. Further research is indicated to investigate the effect of different dopants on the electrical properties of various dielectric and/or ferroelectric thin-films of commercial interest deposited by co-sputtering at elevated temperatures.
